# What do Accredited Social Health Activists need to provide comprehensive care that incorporates non-communicable diseases? Findings from a qualitative study in Andhra Pradesh, India

**DOI:** 10.1186/s12960-019-0418-9

**Published:** 2019-10-22

**Authors:** Marwa Abdel-All, Seye Abimbola, D. Praveen, Rohina Joshi

**Affiliations:** 1 Sydney Medical School, University of Sydney, Sydney, New South Wales Australia; 2 The George Institute for Global Health, University of New South Wales, Sydney, Australia; 3 The George Institute for Global Health, University of New South Wales, New Delhi, India

**Keywords:** Accredited Social Health Activists, ASHAs, Community Health Workers, CHWs, Non-communicable diseases, NCDs, Comprehensive care, LMICs

## Abstract

**Background:**

The Indian National Program for Cardiovascular Disease, Diabetes, Cancer and Stroke (NPCDCS) was introduced to provide non-communicable disease (NCD) care through primary healthcare teams including Accredited Social Health Activists (ASHAs). Since ASHAs are being deployed to provide NCD care on top of their regular work for the first time, there is a need to understand the current capacity and challenges faced by them.

**Methods:**

A desktop review of NPCDCS and ASHA policy documents was conducted. This was followed by group discussions with ASHAs, in-depth interviews with their supervisors and medical officers and group discussions with community members in Guntur, Andhra Pradesh, India. The multi-stakeholder data were analysed for themes related to needs, capacity, and challenges of ASHAs in providing NCD services.

**Results:**

This study identified three key themes—first, ASHAs are unrecognised as part of the formal NPCDCS service delivery team. Second, they are overburdened, since they deliver several NPCDCS activities without receiving training or remuneration. Third, they aspire to be formally recognised as employees of the health system. However, ASHAs are enthusiastic about the services they provide and remain an essential link between the health system and the community.

**Conclusion:**

ASHAs play a key role in providing comprehensive and culturally appropriate care to communities; however, they are unrecognised and overburdened and aspire to be part of the health system. ASHAs have the potential to deliver a broad range of services, if supported by the health system appropriately.

**Trial registration:**

The study was registered with “Clinical Trials Registry – India” (identifier CTRI/2018/03/012425).

## Introduction

During the last decade, many low-income and middle-income countries (LMICs) have invested in community health worker (CHW) programmes due to their distinctive capacity to reach under-served populations [1]. CHWs have improved access to healthcare and led to better outcomes for maternal and child health [2], and for a range of programmes to control infectious diseases like malaria, human immunodeficiency virus (HIV), and tuberculosis (TB). In recent years, there have been research studies evaluating the effectiveness of CHWs to address common non-communicable diseases (NCDs) like cardiovascular disease [3, 4] and mental health [5, 6]. Since most of the evidence is from research and small-scale national programmes [7], it is important to understand how these interventions are implemented and embedded in the routine work of CHWs. It is also essential to understand how the knowledge and skill-set of CHWs is expanded, and workflow streamlined from traditionally focusing on selective vertical programmes to now working across horizontal programmes with a life-course approach.

The female community health workers of India, known as Accredited Social Health Activists (ASHAs), form one of the largest community-based health workforce in the world [8]. Each ASHA supports 1000 people for better access to public health services. In most states of India, ASHAs are volunteers who receive financial compensation for attending training and performance-based incentives to provide specific tasks like immunisation, referral of pregnant women to health centres, and accompanying them for institutional deliveries [9, 10]. Other responsibilities include linking the community to the health system, community sensitization to new initiatives, health education, referral to health centres, and supporting medication adherence for infectious diseases such as tuberculosis through the directly observed treatment short-term programme [11]. They support the auxiliary nurse midwives (ANMs) in delivering maternal and child health-related services.

India has been facing an escalating burden of non-communicable diseases (NCDs) which are is responsible for almost six million deaths (60% of all-cause mortality) annually [12]. Cardiovascular disease, respiratory disease, cancer, and diabetes account for most of the NCD-related deaths, 26% of which occur prematurely between the age of 30 and 70 years [12]. In order to address this growing problem, the Government of India launched the National Program for Cardiovascular Disease, Diabetes, Cancer and Stroke (NPCDCS) in 2010 [13]. NPCDCS was designed to provide comprehensive primary healthcare to communities through the primary health centres (PHCs). The programme aims to prevent and control common NCDs by increasing community awareness, facilitating early detection of undiagnosed cases, and linking the identified cases with the health system for follow-up and continuity of care. An important component of the programme involves capacity building and training of the health workforce including the ASHAs to deliver these interventions.

In order to strengthen health services for NCD control at the community level, there is a need to understand the current capacity, working conditions, and challenges faced by ASHAs—this study aims to achieve these objectives. Furthermore, this study aims to understand the role of the health system to support ASHAs to perform these duties.

## Methods

This study involved two steps: first, a review of the current policies for NCDs management and the ASHA workforce and, second, a qualitative study including focus group discussions with ASHAs and community members and semi-structured in-depth interviews with ASHA’s supervisors and senior medical officers at the district level.

### Study setting

This study was conducted in Guntur, the second most populous district of Andhra Pradesh, in south India. It took place between April and June 2018 in close collaboration with policymakers and key stakeholders of the ASHA programme. Guntur has 85 primary health centres serving around five million population, with two thirds classed as rural. Guntur was selected because its rural-urban distribution and health system is similar to most regions of India. Secondly, The George Institute has its field office and team in the region which enables collaboration with the local government and facilitates data collection.

### Study participants

We purposively selected 41 primary health centres for their geographic spread, encompassing all different serving population capacities and representing all the rural and remote regions of Guntur. We invited five ASHAs from each primary health centre to ensure that a broad range of perspectives are represented. In total, we conducted 13 focus group discussions with 180 ASHAs and five focus group discussions with 47 community members. We also interviewed 13 auxiliary nurse midwives and seven medical officers. In addition, we interviewed two senior medical officers at the district level. Each focus group discussion involved 9–14 participants and lasted approximately 75 min, and each interview lasted approximately 45 min.

### Study instruments

For the interviews and group discussions, we modified the Community Health Worker Assessment and Improvement Matrix (CHW-AIM) [14] to include interventions for the screening and management of cardiovascular diseases and diabetes mellitus based on the World Health Organization’s Package of Essential Non-Communicable Diseases Interventions (WHO PEN) [15] and NPCDCS [13]. The modified version was used as a guide for the qualitative interviews and group discussions. Respondents were asked to reflect on four key areas:

- Current engagement and responsibilities of ASHAs in the NPCDCS
- Support strategies including training, supervision, and reimbursement schemes
- Challenges and needs of ASHAs
- Expanding the role of ASHAs beyond maternal and child health

### Data collection and management

For the document review component of the study, we obtained online all publicly available policy and programme documents relating to the NPCDCS, and ASHA recruitment, training, assessment, remuneration, career progression, supervision, and job description. Data extraction from the policy documents included information on the expected engagement and responsibilities of ASHAs in the NPCDCS, programme governance, and inputs such as remuneration, training, and evaluation schemes. We then identified the system components of the ASHA programme that were most relevant to the knowledge and skills required for NCD prevention and control. These were also used to inform the interviews and group discussion guides.

All in-depth interviews and focus group discussions were conducted in Telugu, the local language, and run by experienced facilitators and note takers who met the participants for the first time for the study. The auxiliary nurse midwives facilitated the invitation of the ASHAs and community members for the focus group discussions which took place within the primary health centres premises. At the beginning of each session, the study objectives were explained to the participants and confidentiality was assured. The facilitators were briefed regularly by two of the authors (MA and RJ) and discussion guide updated accordingly. The focus group discussions and interviews were audio recorded and transcribed verbatim and the data was translated to English.

### Data analysis

Iterative-inductive thematic analysis of the qualitative data was conducted. We identified the different emerging themes, which were then categorised into codes and sub-codes and compared across participant groups to inform the interpretation of the data. We sought to identify how intersections between the ASHA programme and the NPCDCS influences the capacity and disposition of ASHAs to deliver NCD services from the point of view of both the health system and of ASHAs themselves.

### Ethics

Ethical approval was obtained from Ethics Committee of The George Institute for Global Health, India. All participants gave written informed consent in Telugu.

## Results

**Fig. 1:**
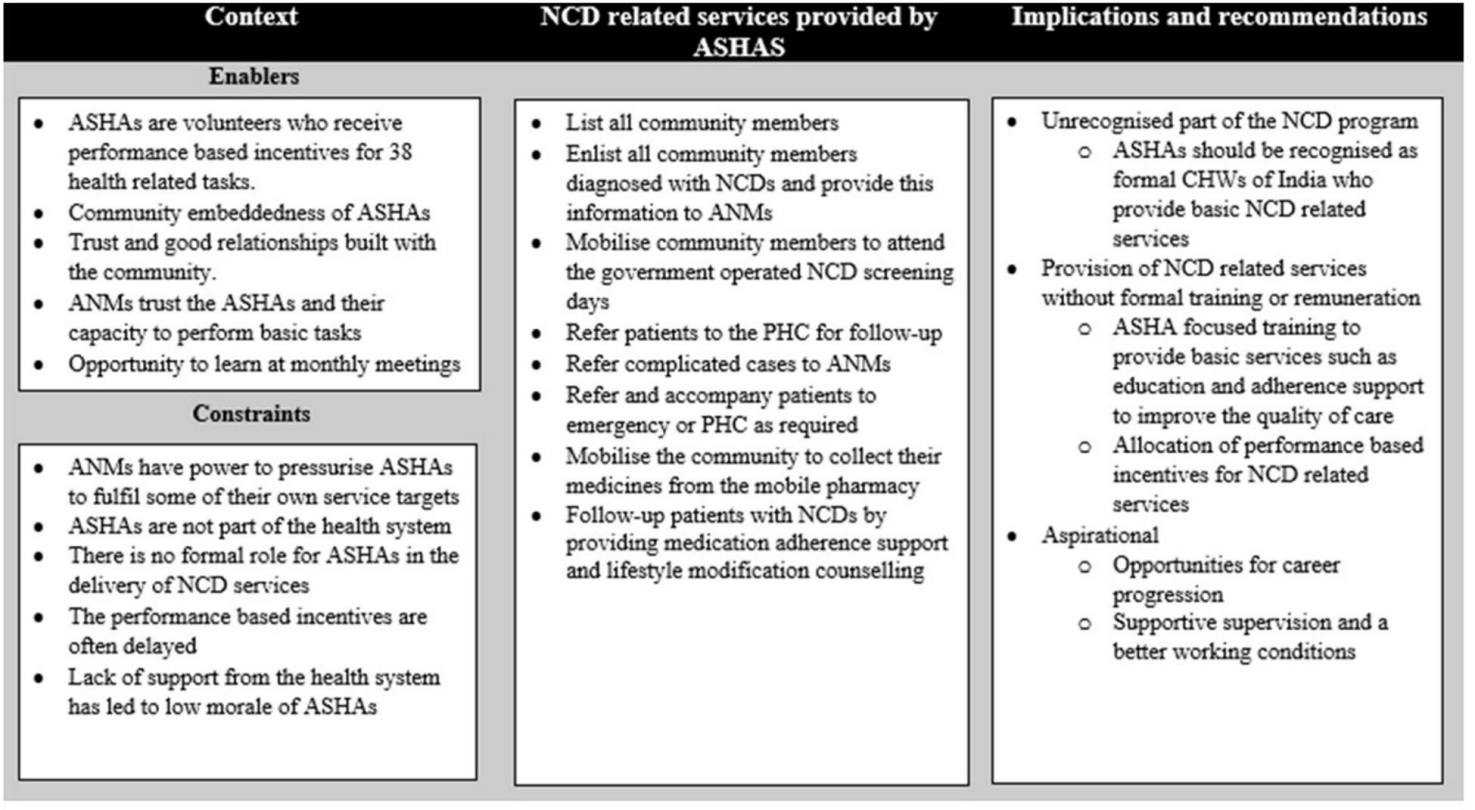
Enablers and barriers in the delivery of the National Program for Cardiovascular Disease, Diabetes, Cancer and Stroke services through the Accredited Social Health Activists

Three themes emerged to characterise how the ASHA programme intersects with the NPCDCS to influence the capacity and disposition of ASHAs towards delivering NCD services. First, while ASHAs are identified as part of the NPCDCS team at the policy level, they are not recognised as part of the formal NPCDCS service delivery team on the ground. Second, ASHAs are overburdened, since they deliver several NPCDCS activities as well as their routine primary care workload without receiving training or remuneration for the NCD activities. Third, they aspire to be formally recognised as employees of the health system. ASHAs are enthusiastic about their work including the NPCDCS responsibility and remain an essential link between the health system and the community. See Fig. 1 for further characterisation of contextual enablers and constraints, activities delivered by ASHAs for NCD care, and the implications.

### Unrecognised: ASHAs are not a formal member of the NPCDCS healthcare team

The NPCDCS policy review identified detailed guidelines about the role of various team members at the primary health centre and sub-centre levels. According to the policy, ASHAs are to perform a broad range of program-related tasks. These entail screening for chronic disease risk factors using “Community Based Assessment Checklist”, surveying the community for family history of NCDs, mobilising the community to attend screening days, improving community awareness of the importance of following healthy lifestyle, and following up patients for medication adherence and continuity of care.

Our study found out that while ASHAs are not officially recognised as members of the NCPDCS services delivery team by the primary care team, they are involved in some NPCDCS activities. Community members discussed that ASHAs advised them about healthy eating and lifestyle to help them with their poorly controlled blood pressure and diabetes. ASHAs also remind the community members to attend the regular screening campaigns and to pick up their monthly medications when necessary. As part of NPCDCs, a NCD training module has been prepared specifically to train ASHAs about NCDs, their risk factors, how to raise community awareness of NCDs, and promoting healthy lifestyles [13]. However, ASHAs in this region have not been trained in this module. In addition, none of the activities described in the NPCDCS policy guidelines are incorporated in the performance-based incentive scheme for ASHAs.

Although ASHAs are recognised as part of the primary care service delivery team, they are not formal employees of the health system. The perception among some of the medical officers interviewed was that ASHAs are not competent and do not have the knowledge and necessary skill-set to provide NCD services. In the words of one of the medical officers, “ASHAs are volunteers who can only perform tasks during their free time and their main role is to link the community to the health system”. Another medical officer added “ASHAs’ basic level of education would not allow them to learn as fast as other technical members of the healthcare team”*.* This has caused ASHAs to experience some harassment and disrespect by members of the primary healthcare team. One of the ASHAs explained “when we take a patient to the hospital, they would tell us “you are an ASHA, stay outside”*.* Patients would react by saying “there is no point for you to accompany us to the hospital” and consequently “they lose their trust in ASHAs”*.* But despite this, one of the senior medical officers highlighted that ASHAs are always considered a part of the health system—in his words: “any new program launched by the government with ASHAs will have their names listed on the program portal along with other healthcare providers”*.*

### Overburdened: ASHAs are the ones on the ground delivering NPCDCS services

The main role of ASHAs is to mobilise their communities to increase health services’ utilisation. Apart from participating in a wide range of primary healthcare programs, they are required to carry out regular household surveys to update the demographic profile of the communities they serve. This role has helped the ASHAs understand their community’s needs, identify individuals requiring different health services, and build trust with the community members. A medical officer described ASHAs as “the first person to know community members’ symptoms”, and one of the senior medical officers added that “community members feel [the] ASHA as their own person … [the] first thought which comes to their mind when they are sick is ASHA”. Counting on this unique status, auxiliary nurse midwives have relied on ASHAs to fulfil some of their own NPCDCS targets. These include reporting previously diagnosed cases of NCDs to auxiliary nurse midwives, inviting community members aged 30 years and above for screening days, and ensuring that patients have collected their monthly medication supply. Most ASHAs reported that they did not receive a job description defining their expected role.

Without formal training on NCDs, ASHAs often need to provide health education regarding nutrition and physical activity for individuals diagnosed with NCDs such as diabetes or high blood pressure. An ASHA noted “we visit the patients at home and check if they are using their medicines and advise them to go for check-ups regularly. Sometime, we give them our phone number and tell them to call us in case of any emergency to help refer them to a nearby hospital”. When asked how they get to know about patient counselling, they mentioned that they often ask auxiliary nurse midwives for specific counselling to respond to community enquiries. Others mentioned that these topics were discussed during the monthly meetings at the primary health centre. One medical officer explained that ASHAs and auxiliary nurse midwives are invited for monthly refresher training meetings at the primary health centre, where different topics are discussed such as cardiovascular disease risk factors and healthy lifestyle promotion. Although these topics are not targeted for ASHAs, they acquired knowledge through these meetings.

Even though ASHAs are volunteers who are expected to work for a couple of hours per day, and receive performance-based incentives, they are overburdened by the additional workload and end up working long hours without receiving any incentives to perform many of these tasks (e.g. medicine adherence support). Several ASHAs have a second job to supplement their income. One ASHA said: “We get pressurised to finish more tasks and put aside our family needs … We end up getting blamed by our families, or even losing the second job we have”. The community members as well expressed their gratitude for the efforts of the ASHAs. One community member said: “ASHAs put in a lot of effort under hard conditions like rainy and hot days …. They provide us with lots of services”*.*

### Aspirational: expectations for the expansion of the role of ASHAs

Stakeholders felt that ASHAs are well placed to help address the growing burden of NCDs at the village level and that ASHAs are enthusiastic about helping people in their communities. The stakeholders acknowledged that ASHAs are interested to be trained and upskilled so that they can support the communities they serve. They also aspire for their role to be recognised by the health system. In the words of one of the senior medical officers: “some ASHAs have completed the auxiliary nurse midwife training themselves … They want to progress their career”. He also noted that the government has shown interest in these more ambitious ASHAs, and there are discussions about career advancement opportunities for ASHAs. Stakeholders emphasised the crucial need for ASHAs to be trained for NCDs. One medical officer said: “it will be very good to train ASHAs for NCDs …. They need to have some knowledge about the different diseases the population is suffering from like diabetes and hypertension which prevents further complications … We can make a better community”. One of the senior medical officers also highlighted the importance of the prerequisite of basic education for recruitment of ASHAs to ensure that they can acquire further skills and knowledge. He said: “there are ASHA workers who have a higher level of education now … If we can train those for screening and follow-up, it will be good and the community will definitely accept them”. Community members welcomed the idea of ASHAs providing basic NCD services, as they would get better access to health services and save on transportation expenses to the primary health centres for problems that can be resolved by the ASHA. One community member said: “everyone from our village will come to her rather than going very far for check-up … It will be convenient if they do it at home”.

Remuneration for ASHAs was extensively discussed as a factor limiting their job satisfaction. All the stakeholders agreed that ASHAs were not being remunerated in accordance to the services they perform. One of the auxiliary nurse midwives explained: “ASHAs have been helping us in multiple programs; they do not get paid for many of the tasks …. They are not getting paid enough in overall, we have been asking for a pay rise for them”. And community members expressed their concern regarding the poor income of ASHAs and how it is insufficient to cover their family needs. One community member said: “ASHAs care a lot about us … they come to visit us at home and tell us about the available health services …. Hopefully they get paid enough to support themselves”. One of the senior medical officers explained that “the remuneration scheme has been increasingly discussed in the governance meetings, and they are considering a suitable reform in the near future.” Various stakeholders were concerned that providing a fixed salary (instead of payment for performance) may negatively affect the quality and quantity of service they provide. While ASHAs expressed their preparedness to receive more training and to acquire additional skills, they stressed the importance of receiving enough remuneration before they can accept further tasks. Other ASHAs also highlighted the idea that if expanding their role would involve longer work hours, it might not be suitable for all ASHAs, especially the ones with family responsibilities. In general, most ASHAs expressed their hope for a government reform in the near future that can provide them with a basic salary as in the case of other states of India.

## Discussion

This study shows that although ASHAs in Guntur are well accepted by the communities they serve, yet, they are not recognised as an integral part of the NPCDCS service delivery team. While NPCDCS is well designed and described on the policy papers, its roll out lacks details and has led to the sub-optimal implementation of the programme through ASHAs at the community level. ASHAs are not officially delegated any NCDs tasks; however, they are asked to provide basic NCD services without specific training or remuneration to fulfil the NCD-related targets. They contribute to their communities and the health system and seek opportunities to upskill and advance their career. All stakeholders agreed that ASHAs are not appropriately utilised by the health system, even though they can provide community-oriented care as part of efforts to address the growing burden of NCDs. They are capable of connecting and relating to their community based on their relationships and understanding of their community’s culture, beliefs, and norms.

Our findings are in line with literature which highlight the support needed for CHWs to play a fundamental role in improving access to healthcare [3, 16]. Previous studies have demonstrated that trained and supervised CHWs can effectively screen individuals at high risk of cardiovascular disease [17], for cardiovascular risk factors [16, 18], and promote healthy lifestyles for primary prevention of NCDs [19]. Similar to the findings of our study, the literature shows that key enablers to optimise CHW programmes include adequate recognition and integration of CHWs into the health system, functional infrastructure, and a clear role description [20]. In order to optimise the efficiency of the ASHA programme, we recommend that the Indian government commits towards the development and capacity building for the ASHA programme for NCD control. This will involve training, monitoring, and support, with evaluation and career development options.

ASHAs need to be trained to effectively deliver NCD services to their communities. There is a gap between community expectations and what CHWs are empowered to do. Training should reflect the job description, position requirements, and needs to include both technical and non-technical competencies. Topics such as communication and problem solving skills to deal with their daily challenge have been shown to improve motivation, and performance of CHWs [21]. The prerequisite level of education of the ASHAs influences their capability to gain more knowledge and skills to be able to provide appropriate care to the community. In addition, higher educated members tend to be more ambitious for career development opportunities and are more enthusiastic to receive training [22]. The recent WHO recommendations for optimising CHW programmes identified community embeddedness, where the community has a sense of ownership of the programme and contributes towards the CHW selection process, as an effective way of sustaining community-CHW relationship and strengthening CHW programmes [23]. Furthermore, ongoing supportive supervision and quality improvement approaches are critical for the effectiveness of CHWs [24]. The literature highlights the consequences of the poor supervision in negatively affecting the performance of the CHWs [25].

While policy decisions in India are made at the national level, there is variation in implementing these policies at the state level. One of the limitations of our study is that it reports findings from only one district in south India. The findings of our study may not be applicable to ASHAs in other states, especially where ASHAs receive a fixed salary and support from their supervisors [26]. Furthermore, cultural, gender, and societal influences differ across India, which have an impact on service delivery at the community level. These differences could not be captured in our study. Furthermore, while this study identifies some of the needs of ASHAs and what may be required to support them to deliver NCD services optimally, our findings do not indicate how policymakers may prioritise which support to provide. The use of other research methods, such as Discrete Choice Experiments, may indeed be necessary to identify what those priorities might be. It is of value to make the best use of the often limited resources of the health systems, to implement contextualised policy interventions that can be attractive to the CHWs, and to help sustain the established programmes [22].

The findings of this study may inform the implementation of few relevant policies announced by the Government of India. The first policy relates to an increment in ASHAs’ performance-based incentives along with social security coverage [27]. More recently, the Government of Andhra Pradesh announced an increase in ASHAs’ salary from 3000 Indian Rupees to 10 000 Indian Rupees per month [28]. These were announced in response to a demand from ASHAs for income commensurate to their workload. The second policy includes strategies to support ASHAs seeking to complete secondary education through the Open School System and that will prioritise them for the auxiliary nurse midwife course [29]. Third, the government has introduced 38 incentivised tasks for ASHAs in 2017 in addition to the routine activities announced in 2014 [30], to provide some level of predictable income; however, none of these activities are NCD service related. While these policies have not yet been implemented, it will be important to measure the impact of these policies over time.

As the range of services provided by ASHAs expands, there will be a debate on whether to increase the number of ASHAs or to create a separate NCD-specific cadre [31]. However, in empowering ASHAs for more responsibilities, knowledge, and skills, it is important that they continue to be embedded in the community so that they develop a strong relationship that is necessary to effectively provide healthcare across the life course. With recent evidence suggesting that there is value in incorporating digital technologies with CHW programmes, India is currently undergoing a digital transformation with an aim to improve health service delivery. This strategy has the potential to support ASHAs via training, decision support, referrals, and follow-up, thereby helping them deliver a broader range of quality health services [32]. However, there is a need for more evidence about how these technologies may support the workforce and strengthen health systems [33].

## Conclusion

This study shows that in addressing the increasing burden of NCDs in LMICs, CHWs are considered particularly appropriate to provide culturally appropriate care among hard to reach populations. CHWs have the potential to deliver NCD services effectively if provided with appropriate training and supervision to maintain quality of service and adequately remunerated to keep them motivated. Community embeddedness, health system recognition, and supporting infrastructure with suitable funding for remuneration schemes are critical for success of CHW programmes. Understanding the needs and motivational factors for CHWs can help improve their performance and improve health outcomes.

## Data Availability

All the data are made publicly available.

## Abbreviations

ASHAs: Accredited Social Health Activists
CHWs: Community Health Workers
LMICs: Low- and Middle-Income Countries
NCDs: Non-communicable diseases
NPCDCS: National Program for Cardiovascular Disease, Diabetes, Cancer and Stroke
